# Supplementary material for: Sexual Dimorphism in Sex Hormone Metabolism in Human Skeletal Muscle Cells in Response to Different Testosterone Exposure
Source: Biology (Basel). 2024 Oct 5;13(10):796. doi: 10.3390/biology13100796 (PMC11504033; doi:10.3390/biology13100796)
Supplement: Supplementary file 1 [file biology-13-00796-s001.zip › biology-3213962-supplementary.pdf]

Table S1. IL6 and IL8 release from 46XY and 46XX human skeletal muscle cells treated with different doses of testosterone. Data are expressed as fold induction *vs.* ctr ± SD. T=testosterone. Arrows represent the percentage of increment or decrement in comparison to the untreated condition (ctr). *p<0.05, **p<0.01 *vs.* ctr.

**Myokines release (fold induction *vs.* ctr) ± SD**						
			**46XY**			
**T (nmol/L)**	**IL6**	↑↓	% of increment/ decrement	**IL8**	↑↓	% of increment/ decrement
**0.5**	1.2±1.0	↑	60%	1.2±0.0	↑	8%
**2**	1.0±0.3	↑	140%	1.0±0.0	↑	5%
**5**	1.4±0.4**	↑	210%	1.3±0.0	↑	13%
**10**	1.3±0.5**	↑	230%	1.3±0.2*	↑	20%
**32**	1.4±0.4**	↑	320%	1.4±0.0*	↑	40%
**100**	1.2±0.2**	↑	120%	1.2±0.0*	↑	30%
			**46XX**			
**0.5**	1.3±0.0*	↑	30%	1.3±0.0	↑	30%
**2**	0.5±0.0**	↓	30%	0.5±0.1**	↓	40%
**5**	0.4±0.0**	↓	50%	0.4±0.0**	↓	60%
**10**	0.3±0.0**	↓	70%	0.3±0.0**	↓	30%
**32**	0.2±0.0**	↓	70%	0.2±0.0**	↓	80%
**100**	0.0±0.2**	↓	90%	0.1±0.0**	↓	99%
